# Current Trends in RNA Virus Detection via Nucleic Acid Isothermal Amplification-Based Platforms

**DOI:** 10.3390/bios14020097

**Published:** 2024-02-11

**Authors:** Le Thi Nhu Ngoc, Young-Chul Lee

**Affiliations:** 1 Department of Nano Science and Technology Convergence, Gachon University, 1342 Seongnam-Daero, Sujeong-gu, Seongnam-si 13120, Gyeonggi-do, Republic of Korea; nhungoc@gachon.ac.kr; 2 Department of BioNano Technology, Gachon University, 1342 Seongnam-daero, Sujeong-gu, Seongnam-si 13120, Gyeonggi-do, Republic of Korea

**Keywords:** RNA virus detection, loop-mediated isothermal amplification (LAMP), recombinase polymerase amplification (RPA), recombinase-aided amplification (RAA), nucleic acid sequence-based amplification (NASBA), point-of-care testing (POCT)

## Abstract

Ribonucleic acid (RNA) viruses are one of the major classes of pathogens that cause human diseases. The conventional method to detect RNA viruses is real-time quantitative reverse transcription polymerase chain reaction (qRT-PCR), but it has some limitations. It is expensive and time-consuming, with infrastructure and trained personnel requirements. Its high throughput requires sophisticated automation and large-scale infrastructure. Isothermal amplification methods have been explored as an alternative to address these challenges. These methods are rapid, user-friendly, low-cost, can be performed in less specialized settings, and are highly accurate for detecting RNA viruses. Microfluidic technology provides an ideal platform for performing virus diagnostic tests, including sample preparation, immunoassays, and nucleic acid-based assays. Among these techniques, nucleic acid isothermal amplification methods have been widely integrated with microfluidic platforms for RNA virus detection owing to their simplicity, sensitivity, selectivity, and short analysis time. This review summarizes some common isothermal amplification methods for RNA viruses. It also describes commercialized devices and kits that use isothermal amplification techniques for SARS-CoV-2 detection. Furthermore, the most recent applications of isothermal amplification-based microfluidic platforms for RNA virus detection are discussed in this article.

## 1. Introduction

Deoxyribonucleic acid (DNA) and ribonucleic acid (RNA) are the most important molecules in cell biology. DNA encodes the instruction manual for life. It carries and stores genetic information, which encodes the amino acid sequence responsible for cell functions. RNA molecules are chemically similar to DNA and are important in transmitting genetic information for protein synthesis [1]. Moreover, RNA viruses are challenging because they can mutate rapidly, leading to genetic diversity [2]. It is crucial to detect RNA viruses quickly and accurately to diagnose infectious diseases promptly and manage outbreaks effectively [3,4]. Traditional methods of RNA virus detection often require complex and time-consuming processes, making it difficult to respond promptly [5]. For instance, polymerase chain reaction (PCR) is the gold standard for nucleic acid amplification-based assay for diagnosis. Despite its broad applicability, PCR application remains limited to highly equipped laboratories [6]. The need for sophisticated and expensive thermocyclers makes it challenging to use PCR in point-of-care testing (POCT) analysis.

Recently, nucleic acid isothermal amplification-based platforms have emerged as powerful tools for sensitive, specific, and rapid detection of RNA viruses [7,8,9]. These innovative techniques circumvent the need for thermal cycling, characteristic of PCR-based methods, by enabling DNA amplification at a constant temperature [7,8,9]. Isothermal amplification assays offer several advantages, such as simplifying instrumentation, reducing assay time, and increasing accessibility, making them ideal candidates for POCT and resource-limited settings [10]. This study explores the advancements and applications of nucleic acid isothermal amplification-based platforms in the context of RNA virus detection. We delve into the diverse methodologies, including loop-mediated isothermal amplification (LAMP) [11,12], recombinase polymerase amplification (RPA) [13,14], recombinase-aided amplification (RAA) [15,16], nucleic acid sequence-based amplification (NASBA) [17,18], and helicase-dependent amplification (HDA) [19,20], elucidating their principles, key components, and amplification mechanisms. This review focuses on the most common isothermal amplification methods, including LAMP, RPA, and RAA. Additionally, we discuss the strengths and limitations of each technique, along with recent innovations and optimizations aimed at enhancing sensitivity, specificity, and user-friendliness.

Through a comprehensive examination of these cutting-edge technologies, this study seeks to contribute to the ongoing efforts to advance RNA virus detection methodologies. We aim to accelerate progress toward efficient, point-of-need diagnostics that significantly impact disease surveillance, outbreak management, and global public health by harnessing the potential of nucleic acid isothermal amplification-based platforms.

## 2. Loop-Mediated Isothermal Amplification 

### 2.1. Principle of LAMP for RNA Virus Detection

The LAMP method was first proposed by Notomi et al. in 2000 to improve the specificity and sensitivity of amplification [21]. LAMP has been widely employed in diagnostic fields to realize POCT devices because of their high specificity, sensitivity, accuracy, and simplicity [22]. LAMP synthesizes new DNA strands based on the strand-displacement activity of Bst polymerase. The enzyme catalyzes nucleic acid amplification at approximately 60–65 °C. The process requires more than two primer sets to recognize six different sites of the target gene. Four major primers for LAMP include forward and reverse outer primers (F3 and B3) and forward and reverse inner primers (FIB and BIP). Additionally, a loop primer (forward loop primer (LF) or backward loop primer (LB)) is usually employed to increase the reaction speed [21,22]. The final products of LAMP stem-loops DNA have different repeated target sequences. 

LAMP products can be detected using a variety of approaches, including sequence-independent and sequence-dependent methods. Regarding sequence-independent methods, turbidity monitoring and gel electrophoresis are commonly applied [23]. However, owing to the need for sophisticated instruments and technical expertise, the application of turbidity and gel electrophoresis has been replaced by other simple and efficient methods, such as using metal indicators, including calcein or hydroxynaphthol blue. Their applications are based on the formation of byproducts during LAMP reactions. The obtained results are either fluorescent or colorimetric signals obtained using calcein or hydroxynaphthol blue, respectively. Another approach to detecting LAMP products is using pH indicators to indicate the ion produced during the amplification process. Phenol red is commonly used in LAMP mixtures with low buffer concentrations [23]. In addition to these indirect methods, LAMP amplicons can be evaluated directly using intercalators such as Sybr Green I or DNA dyes such as crystal violet. However, sequence-independent methods sometimes lead to false negative signals due to primer-dimer issues. 

Sequence-dependent methods can address the problems of sequence-independent methods. Sequence-dependent methods include homogeneous and heterogeneous methods [23]. They are further categorized into particle-based and particle-free methods, which are then classified according to the sensing techniques and transducing elements used. First, homogeneous methods detect LAMP products based on signals generated and detected from the entire reaction mixture volume. During signal transduction, biological recognition elements are homogeneously distributed in the reaction volume, immobilized on suspended particles (particle-based detection), or dissolved in solution (particle-free detection). These homogeneous detection methods are advantageous because they provide real-time or immediate results without needing post-amplification processing. Conversely, heterogeneous detection methods involve separating the LAMP products from the reaction mixture and detecting them using various assays or techniques [23]. The methods use planar sensor surfaces connected to a transducer element. Then, biorecognition elements that capture the target analysis are near or are bonded to the sensor surface to detect the signal. The following classification level is based on the differentiation of the signal transduction and sensing techniques. Signal transduction can be categorized according to the transducing element, including optical (fluorescence, chemiluminescence, and colorimetric), magnetoresistive, magnetic, piezoelectric, and electrochemical [23]. 

### 2.2. Applications of LAMP for RNA Virus Detection

Recently, LAMP has been widely applied in diagnostic clinical samples (Table 1). This section briefly describes the recent achievements of LAMP in detecting RNA viruses using different detection methods, such as fluorescent, colorimetric, and electrochemical methods. 

#### 2.2.1. Fluorescence-Based Detection Methods

A single-tube assay was developed to detect SARS-CoV-2 in clinical samples. RT-LAMP was combined with clustered regularly interspaced short palindromic repeats (CRISPR) and CRISPR-Cas12a to realize sensitive fluorescent signals [34]. The proposed strategy increased the sensitivity and specificity of the RT-LAMP assay. In addition, the closed-tube system reduced the risk of contamination that can occur in other LAMP assays. A variant-tolerant diagnostic test was developed to overcome the limitations of the general molecular test for diagnosing SARS-CoV-2 variants [34]. The approach combined a LAMP assay using high-fidelity DNA polymerase and a high-fidelity DNA polymerase-medicated probe. The probe, which has the same sequence as a loop primer, forms a dumbbell-shaped structure where the fluorescent signal is quenched. High-fidelity DNA polymerase can cleave the probe, releasing a fluorescent signal and generating the specific fluorescent signal of the multiplex real-time LAMP assay. This strategy offered potential solutions for detecting highly variable RNA viruses. 

A SlipChip device has been proposed for detecting SARS-CoV-2 in a quantitative manner [45]. The SlipChip creates droplets of varying sizes to enable digital TR-LAMP, a method for detecting SARS-CoV-2 nucleic acid [45]. This device provides an accessible POCT assay for rapid detection of SARS-CoV-2, and a three-dimensional cartridge connected to a smartphone reader was developed for real-time RT-LAMP. The system tested clinical samples and showed results within 30 min with an LOD of 50 RNA copies [46]. However, it is important to note that although LAMP is a powerful tool for SARS-CoV-2 screening, it may produce a false positive signal [36]. Therefore, an optimized fluorescent assay, termed LATERN, was developed to minimize spurious amplicons of LAMP. The assay took only 30 min to analyze swab and saliva samples with an LOD of 8 copies/reaction. 

A portable device was developed to detect the Mayaro virus in a from-sample-to-answer manner [28]. The Mayaro virus is a mosquito-borne virus that causes severe diseases with symptoms similar to those caused by the Dengue or Zika viruses. Therefore, an effective tool to diagnose this virus is vital for effective treatment. This portable device integrates the whole nucleic acid amplification-based method, allowing for a from-sample-to-answer assay. The device uses a sample preparation tool that carries virus lysis products without pipetting, making it easier and less time-consuming. Additionally, a real-time amplification device is utilized for amplification and detection. The introduced platform detected the Mayaro virus in blood samples in less than 13 min [28]. 

**Figure 1:**
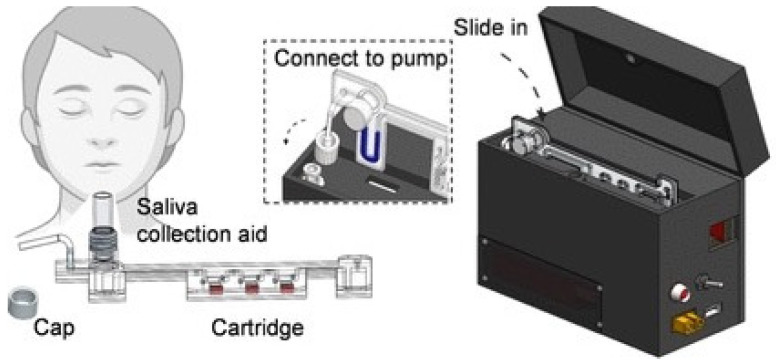
Schematic illustrating saliva-based SARS-CoV-2 self-testing using RT-LAMP on a mobile device. Reprinted with permission from ref. [43]. Copyright (2022) American Chemical Society.

A mobile device was developed to detect SARS-CoV-2 rapidly using RT-LAMP [43]. In this system, a from-sample-to-answer process, including virus preparation, sample dispensing, RT-LAMP, and real-time detection, is automatically performed. The device detected the target in a saliva sample within 45 min with a low LOD of 5 copies/µL (Figure 1) [43]. Fluorescent detection methods can quantitatively detect targets; moreover, fluorescent detection strategies using probes can minimize the possibility of false negative signals and increase sensitivity. However, applying fluorescent approaches using portable devices requires extra equipment like UV sources for signal readout.

#### 2.2.2. Colorimetric-Based Detection Method

Colorimetric detection provides a simple and equipment-free signal readout for LAMP detection. The RT-LAMP method has also been employed to develop devices for RNA virus detection. Notably, a real-time colorimetric LAMP-based device has been created with remarkable success in identifying respiratory viruses [47,48]. The use of magnetic beads to extract nucleic acids from the sample, combined with the microfluidic device designed with an eight-channel array, allowed for rapid and highly sensitive detection of respiratory viruses. Using this device, researchers successfully achieved specific detection of influenza A viruses, including H1N1, H3N2, H5N1, H7N9, influenza B, and human adenoviruses. Moreover, this study introduced a real-time virus detection strategy using colorimetry. The colorimetric detection instrument monitored the amplification process by calibrating an amplification curve based on the change of color of the reaction from purple to sky blue [47]. A self-driven microfluidic device was developed to detect influenza viruses via RT-LAMP. Magnetic beads conjugated with H1N1 aptamers were used to isolate the virus. After the virus lysis step, nucleic acids were amplified using RT-LAMP. Finally, the results were obtained by observing the color change of metal indicators from purple to sky blue. This process took less than 40 min with an LOD of 3 × 10^−4^ hemagglutinating units/reaction [48]. 

A microfluidic device featuring RT-LAMP was introduced to detect SARS-CoV-2. The device targets and measures three genes, including the nucleocapsid gene, the envelope gene, and the RNA-dependent RNA polymerase gene. The device has three main components: a microfluidic platform, a fluidic control module, and a temperature controller. The whole process took approximately 90 min, from sample preparation to detection. This approach offered a novel strategy for detection and quantification on a platform with a high specificity and sensitivity [49]. 

**Figure 2:**
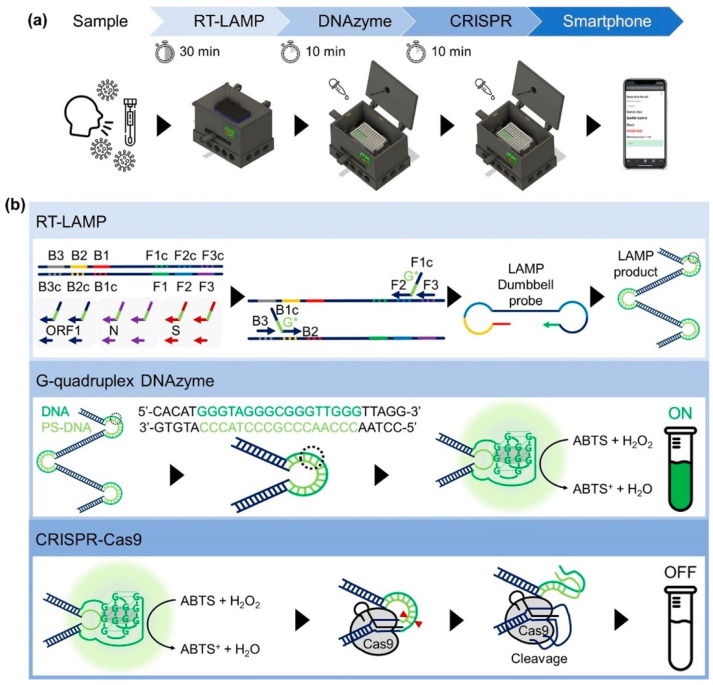
Schematic illustrating (**a**) the procedure of the DAMPR assay for SARS-CoV-2 detection; (**b**) principle of the DAMPR assay including RT-LAMP, G-quadruplex DNAzyme, and CRISPR-Cas9 reactions. Reprinted with permission from [33]. Copyright (2022) American Chemical Society.

Colorimetric methods are more straightforward and user-friendly than fluorescent approaches; however, they suffer from false positive signals due to spurious amplicons. In addition, indirect detection methods monitor LAMP reactions based on byproducts such as pH change and pyrophosphate, which can lead to false signals. A colorimetric DNAzyme reaction triggered by LAMP and CRISPR, termed DAMPR, was proposed to overcome these issues. This method, using CRISPR-Cas9, eliminated false positive signals from the LAMP assay, thereby increasing the accuracy and sensitivity to attomole within 1 h [33] (Figure 2).

#### 2.2.3. Others Detection Methods

**Figure 3:**
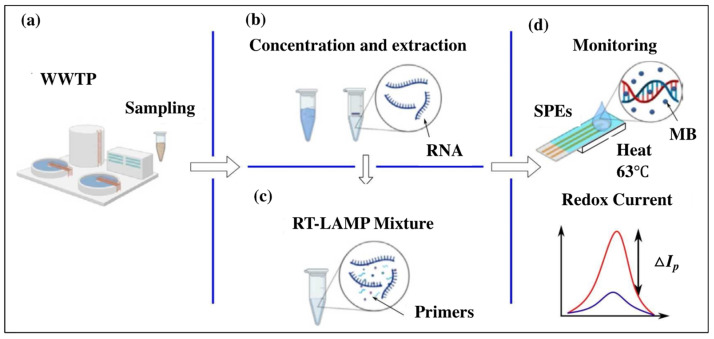
Workflow of RT-LAMP-based electrochemical sensor in wastewater samples: (**a**) sampling from wastewater treatment plant; (**b**) nucleic acid extraction and concentration; (**c**) RT-LAMP mixtures for genetic amplification; (**d**) electrochemical monitoring of the RT-LAMP products via redox current. Reprinted with permission from ref. [44].

There are many methods for detecting nucleic acid amplification products, including optical, colorimetric, or electrochemical methods. Electrochemical approaches offer practical tools for detection with many advantages, including a low cost, portability, high reliability, and miniaturization. An electrochemical sensor was proposed to detect SARS-CoV-2, which offers many functions, including wastewater sampling, RNA preconcentration, RT-LAMP, and electrochemical detection of LAMP products [44]. For electrochemical monitoring, methylene blue was utilized as a redox intercalator. During testing with a wastewater sample, the sensor demonstrated exceptional performance, and the entire testing process took only about 2 h, producing highly quantitative results (Figure 3). A novel hydrogen ion-selective RT-LAMP sensor was proposed to detect HIV-1 RNA efficiently [50]. The device employed hydrogen ions as a signal-producing compound and detected HIV-1 RNA in real human serum within 25 min. The LOD of the assay was 10 copies per tube for clinical samples.

## 3. Recombinase Polymerase Amplification

### 3.1. Principle of RPA

RPA is a nucleic acid amplification technique used for isothermal amplification of DNA and RNA. RPA is conducted in a single tube and functions similarly to PCR; however, RPA has several key differences that make it useful in certain situations, especially when rapid amplification of nucleic acids is required without the need for a thermal cycler. RPA was first introduced by Piepenburget et al. in 2016 [51]. The method realizes isothermal amplification using polymerase combined with recombinase, single-stranded binding protein, and two primers. Recombinase separates the dsDNA. The hybridization of the primer with the homologous sequence in the target gene is catalyzed by recombinase to form a complex. The complex scans along the DNA length and promotes strand exchange. Single-strand binding proteins stabilize the structure, and polymerase catalyzes the primer extension reaction. This method produces millions of copies in less than 1 h at low temperatures of around 35–40 °C [52,53,54]. 

For the optical formation of recombinase/primer filaments, RPA primers are usually 30–35 bases long, while long primers (e.g., more than 45 bases) are not recommended. Although RPA can amplify long sequences (~1.5 kb), better results can be obtained using shorter amplicons (80–400 bd) [55]. There is no requirement for the melting temperature of RPA primers because primer annealing and elongation are enzyme-mediated and not temperature-driven. 

RPA usually uses two probes: RPA-exo or RPA-fpg [55]. RPA-exo probes enhance the specificity of the RPA-exo reaction by providing a more robust and precise means of detecting target nucleic acid sequences. These probes resist degradation by adding exonuclease activity to the RPA-exo reaction to remove non-specific or mismatched primers and nucleotides. The RPA-exo is a long oligonucleotide containing 46–52 bases, carrying an internal base analog located between the fluorophore and the quencher with the 3′ end blocked. In contrast, the RPA-fpg probe was shorter (32–35 bases) and unsuitable for primer use. The RPA-fpg probe has a 5′ quencher and a fluorophore (5–6 bases) downstream attached to the essential nucleotide’s ribose via a C-O-C linker. The RPA-fpg probe emits a fluorescent signal upon binding to the target sequence, which can be detected and quantified in real-time to monitor the amplification process.

Detecting RPA products involves using various methods to visualize or quantify the amplified DNA or RNA [13]. For example, lateral flow strip methods can rapidly display the results in a visual read-out format. This assay often uses three oligonucleotides (one probe and two primers) and the Twist-Amp^®^ nfo kit. In this method, capture probes bound to the test line of the strip will capture the amplified DNA or RNA, producing a visible signal (e.g., a line or color change) if the target is present [13]. Second, agarose gel electrophoresis is widely used to visualize amplification products. After the RPA reaction, the amplified products are run on an agarose gel, and the stained gel reveals the presence and size of the amplicons [13,56]. Third, real-time fluorescence detection is a precise method for monitoring RPA amplification. Fluorescent probes (e.g., FAM, HEX, or Cy5) can be added to the RPA reaction mix. The fluorescence signal increases as the target DNA is amplified. This method allows for quantitative measurements and can be performed using specialized real-time PCR instruments or portable isothermal amplification devices [57]. Fourth, the hybridization-based detection method involves using specific probes labeled with fluorescence or other detectable tags. These probes can be designed to hybridize with the RPA amplicons. Detection can be achieved through fluorescence microscopy, lateral flow, or other methods, depending on the specific design [13]. Finally, digital droplet PCR (ddPCR) can be used to detect RPA products quantitatively [58]. This method involves partitioning the sample into thousands of individual droplets, each containing a specific volume of the reaction mix. It allows for precise quantification of target nucleic acid molecules.

### 3.2. Applications of RPA for RNA Virus Detection

#### 3.2.1. Fluorescence-Based Detection Methods

Integrating RPA with microfluidic devices has the potential to facilitate the use of nucleic acid detection assays at the POC with great simplicity and easy operation [59]. MiCaR, a nucleic acid testing platform, was proposed to detect nine HPV subtypes. This microfluidic platform integrates with multiplex RPA and CRISPR-Cas12a and analyzes samples in only 40 min, with an LOD of 0.26 attomole. The device demonstrated high sensitivity (97.8%) and specificity (98.15) when tested using clinical samples and was able to detect eight common respiratory viruses [60]. A “diagnostics-in-a-suitcase” was proposed for rapid detection of avian influenza A (H7N9) virus [61]. The suitcase contained all the reagents and devices for performing an RT-RPA assay to recognize two genes, hemagglutinin (H) and neuraminidase (N), of the H7N9 virus. Highly sensitive detection of 10 and 100 RNA molecules was achieved within 2–7 min. The system is user-friendly, portable, and can be operated by a solar-powered battery, making it ideal for use in low-resource areas. 

**Figure 4:**
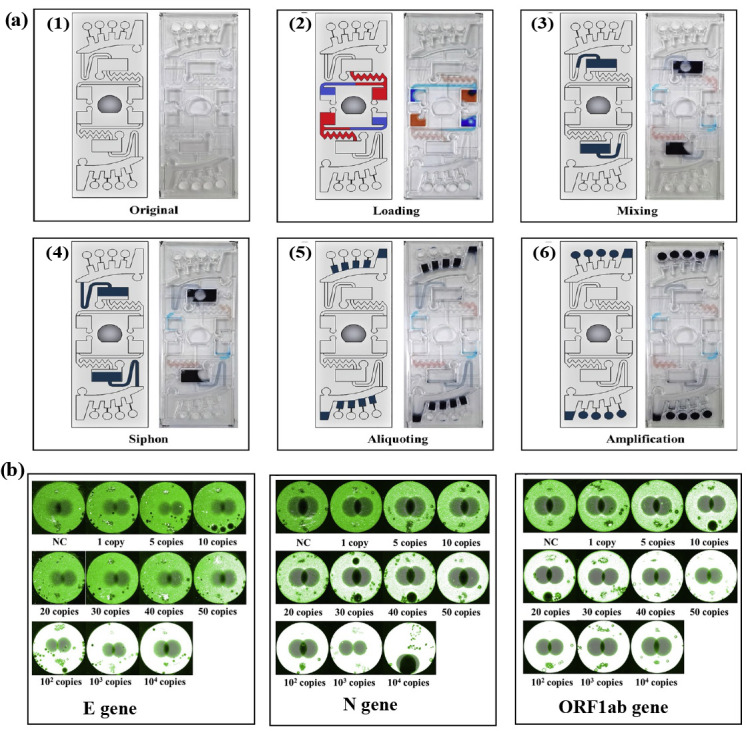
(**a**) Schematic illustration of the flow control of the centrifugal microfluidic device. (**1**) The device started with the dried RT-RPA reagents, primers, and probes. (**2**) A red dye buffer mixture and blue dye sample mixture were injected to their respective chambers. (**3**) The buffer mixture and the sample mixture burst into the mixing chamber, and then were mixed in both counterclockwise and clockwise directions. (**4**) The siphon finished priming via capillary reaction. (**5**) The mixture was transferred into the aliquoting chamber. (**6**) The mixture burst from the aliquoting chamber into the reaction chamber. (**b**) Sensitivity test results of an on-chip RT-RPA for the E, N, and ORF1ab genes of SARS-CoV-2 [62].

A centrifugal microfluidic platform was proposed to detect SARS-CoV-2 rapidly [62]. A multiplex assay targeting the E, N, and ORF1ab genes of SARS-CoV-2 was realized using RT-RPA and fluorescence monitoring. The analysis mostly took place using chips, eliminating the possibility of contamination and human error. However, the proposed platform had several limitations. Firstly, sample preparation was performed off-chip. In the future, integrating sample preparation on a chip platform should be performed. Secondly, high-throughput screening for targets remained limited [62]. Figure 4 illustrates the strategy for using centrifugal microfluidic devices to detect multiple SARS-CoV-2 genes.

Another advantage of RPA is that it can be combined with other isothermal techniques, such as LAMP, to increase sensitivity and decrease false-positive signals [59,63]. A novel one-pot solid-phase RPA coupled with a CRISPR-based system was developed for multiplex detection of SARS-CoV-2 genes [64]. In this approach, solid-phase RT-RPA, where the primers are pre-immobilized on a surface, was employed. The method improved some drawbacks of conventional RT-RPA, such as primer dimers and the production of random byproducts. The proposed system provided a simple, multiplex, and sensitive detection tool for SARS-CoV-2 with an LOD of 20 copies. 

**Figure 5:**
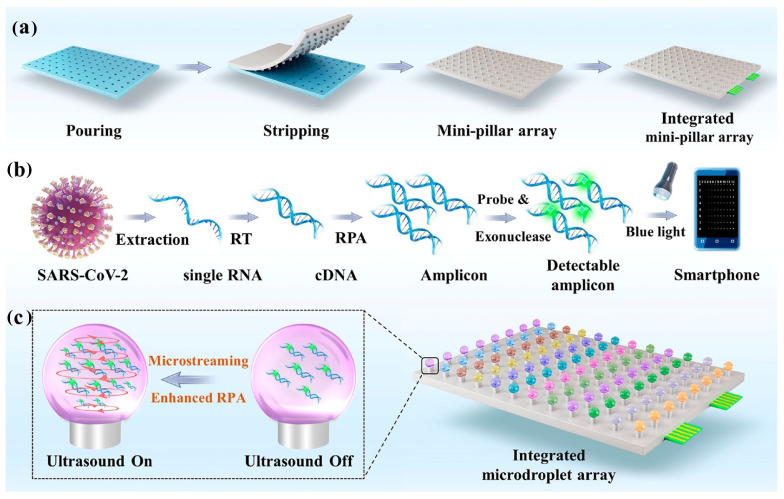
Illustration of the integrated microdroplet array platform for ultrafast and high-throughput diagnosis of SARS-CoV-2: (**a**) design and fabrication of the platform; (**b**) schematic of the RPA react reaction-based flow; (**c**) increase in the interaction between RPA components using ultrasound-driven microstreaming, enhancing the process of RPA. Reprinted with permission from ref. [65]. Copyright (2022) American Chemical Society.

An integrated microfluidic array was proposed for high-throughput detection of SARS-CoV-2 [65]. The platform comprised an ultrasonic unit and minipillar array to perform ultrafast and sensitive RT-RPA. The minipillar was employed here to anchor the microdroplet for the RPA microreactor, which helped reduce reagent and sample consumption. Meanwhile, ultrasound-driven microstreaming was employed for controlled contactless mixing and dispersion in the microreactor. The system detected SARS-CoV-2 in less than 12 min, with an LOD of 0.42 copies (Figure 5) [65]. 

A platform was developed to detect biomarkers for infectious diseases, including SARS-CoV-2 [66]. The approach combined a heterogeneous sandwich-type sensing platform with RPA to detect specific biomarkers using a DNA aptamer, termed H-sandwich. Once the antigens were targeted, the aptamers bound to the antigens, which were then amplified into double-stranded DNA through RPA. The amplification process generated a fluorescence signal that was specific and ultrasensitive, with an attomolar-level sensitivity [66]. Other achievements in using RPA to detect RNA viruses are presented in Table 2. 

#### 3.2.2. Colorimetric-Based Detection Methods

Numerous colorimetric-based detection methods have been developed to detect RNA viruses, including SARS-CoV-2 and other RNA viruses, with a high sensitivity and specificity. A single, one-pot, two-stage POCT method (Penn-LAMP) employing RPA and LAMP was proposed, which achieved a high sensitivity for SARS-CoV-2 detection in an instrument-free manner. The reaction was conducted in a closed tube to avoid contamination risks [82]. Additionally, an all-in-one RPA-CRISPR-Cas12a approach was integrated into a portable device (hippo-CORDS) for the detection of several viruses [72]. The system, termed CORDSv2, improved the assay’s sensitivity by balancing the relationship between RPA amplicons and Cas12a cleavage. Visual detection was achieved with a low LOD of 0.6 copies of DNA and RNA viruses. 

**Figure 6:**
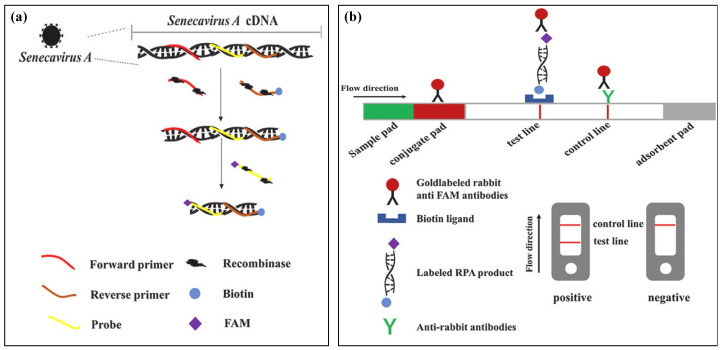
Schematic diagram of the RPA reaction product detection using a lateral flow dipstick. (**a**) RPA-based generation of a specific amplicon for detection using a lateral flow (LF) assay. (**b**) Read-out of the RPA via LF. Reprinted and modified with permission from ref. [77].

A highly sensitive colorimetric assay for detecting SARS-CoV-2 was proposed, which employed RT-RPA coupled with a CRISPR-Cas12a assay and DNA-modified gold nanoparticles for the colorimetric readout [83]. The strategy increased the sensitivity and specificity for detecting SARS-CoV-2 with an LOD of 1 copy/reaction. Moreover, lateral flow assays coupled with RT-RPA for RNA virus detection were developed for on-site detection of RNA viruses, including dengue virus and Senecavirus A infection [74,77,81]. Dengue virus-1, -2, -3, and -4 were detected in 120 clinical samples with LOD of 10 copies of RNA molecules in low-resource areas [81]. Senecavirus A infection was also detected in 25 min at 35 °C with an LOD of 10 copies [77]. The result was directly visualized using a dipstrip in a rapid and user-friendly manner (Figure 6). These assays offer simple, rapid, and user-friendly approaches for detecting RNA viruses. 

## 4. Recombinase-Aided Amplification 

### 4.1. Principle of RAA

RAA is a recent isothermal amplification method that employs three main amplification components: DNA polymerase, recombinase, which is usually extracted from fungi or bacteria, and single-stranded DNA binding protein (SSB) [62,84]. Recombinase proteins (e.g., RecA or a recombinase from a T4 bacteriophage) play an essential role in RAA. These enzymes facilitate the strand exchange and DNA recombination steps in amplification. The SSB proteins stabilize single-stranded DNA regions during the amplification process. DNA polymerase with strand-displacement activity synthesizes new DNA strands during amplification. In addition, RAA uses two specific primers (a forward primer and a reverse primer) designed to target the DNA sequence of interest [85]. These primers are typically labeled with fluorophores and quenchers to allow for real-time monitoring of the amplification process. The principle of this method is similar to RPA [86]. Amplification can be achieved at low temperatures (~37 °C) with a high speed (~30 min). The combination of three main factors in RAA can replace the thermal cycle in PCR for the chain-breaking process. The product of RAA is typically detected using various methods, including visual inspection, real-time fluorescence detection, electrochemical detection, lateral flow assays, turbidity measurements, and gel electrophoresis. Real-time fluorescence-based detection is often preferred for quantitative analysis, while lateral flow assays are functional for rapid and qualitative testing in resource-limited settings. With its simple process, low reaction temperature, and several advantages (speed, precision, and convenient operation), RAA is a promising candidate for on-chip integration [84,87,88]. 

### 4.2. Applications of RAA for Virus Detection

RAA is a recent isothermal amplification technique employing SSB protein, DNA polymerase, and recombinase UX. Compared with LAMP, RAA is simpler and has a lower reaction temperature [62,84]. Recently, many microfluidic platforms have employed RAA to detect RNA viruses (Table 3). 

The RT-RAA method was used to detect Norovirus GII.4, an RNA virus that causes food poisoning, with a detection time of just 30 min at 39 °C. The approach offered promising tools for rapidly screening pathogens in the diagnostic field [92]. 

**Figure 7:**
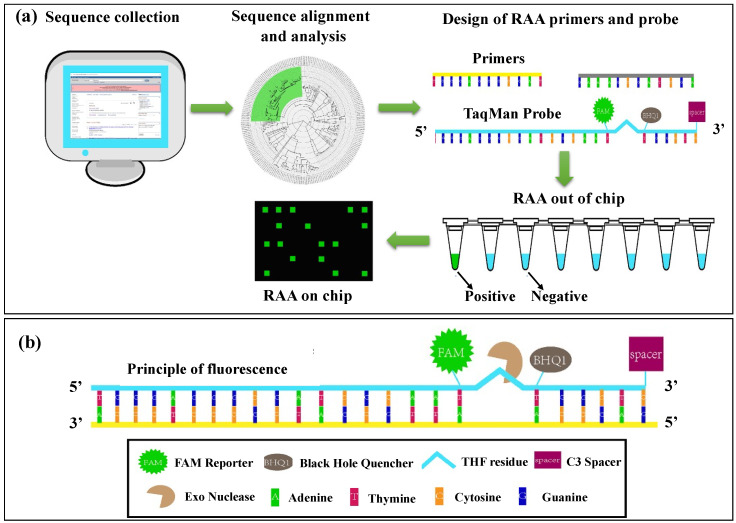
Principle of norovirus digital nucleic acid detection. (**a**) Five key steps of method establishment: sequence collection, sequence alignment and analysis, design of primers and probe, RAA out of chip, and RAA on chip for the quantification process. (**b**) Schematic representation of the RAA probes followed by the generation of positive fluorescence signals. Reprinted and modified with permission from ref. [90].

Another group developed a novel RAA-based microfluidic chip for rapid diagnosis of norovirus (Figure 7) [90]. The microfluidic chip had three layers (PDMS cover, functional, and supporting layers) and could contain a volume of up to 1.2 nL. The chamber digital RT-RAA (cdRT-RAA) was completed within 20 min at 39 °C and showed a good LOD of about 1.02 copies/μL compared to RT-qPCR. This technique exhibited a high robustness and sensitivity and provided an alternative method for the early detection of norovirus. 

An RT-RAA/LFD platform was developed to detect SARS-CoV-2 rapidly, integrating RT-RAA with a lateral flow dipstick to target the nucleocapsid (N) gene of SARS-CoV-2 [93]. For clinical assessment, 100 clinical samples were detected via RT-qPCR and RT-RAA/LFD methods. The RT-RAA/LFD was conducted at 39 °C within 30 min and offered an LOD of 1 copy/μL without cross-reacting with other clinical respiratory pathogens [93]. Furthermore, a CRISPR-Cas13a-assisted immunocapture magnetic bead-enhanced electrochemical biosensor was proposed to detect SARS-CoV-2 rapidly [91]. For target amplification, the RAA reaction was conducted at 42 °C for 30 min. The proposed biosensor provided an ultrasensitive ability as low as 1.66 aM, and different types of viruses (e.g., human coronaviruses, respiratory syncytial virus, influenza A and B virus, and hepatitis B virus) were tested to prove the specificity of the platform. The result was obtained in less than 30 min, with a detection limit of 1 copy/µL [93]. The outstanding features of this device were attributed to low-cast and immobilization-free carbon electrodes, which were utilized to measure the electrochemical signal, and streptavidin-coated immunocapture magnetic beads, which can reduce noise signals and enhance the detection capacity. 

The Hepatitis C virus (HCV) is an RNA virus affecting global public health. An RT–RAA-based platform was proposed to rapidly identify HCV from 46 anti-HCV antibody-positive samples, with a detection limit of 10 copies/µL in just 30 min [94]. However, the platform still has some limitations, such as nucleic acid extraction from the virus being conducted on-tube, and no integration of sample preparation on one platform. Future studies should combine all nucleic acid analysis steps on one platform to ensure increased applications of the developed platform in low-resource areas.

## 5. Other Isothermal Amplification-Based Platforms for RNA Virus Detection

### 5.1. Nucleic Acid Sequence-Based Amplification

NASBA is an efficient technique for RNA amplification and detection. The amplification method operates at a predefined temperature (~41–45 °C) without the aid of a thermal cycler [95]. Three enzymes participate in this isothermal reaction, including avian myeloblastosis virus reverse transcriptase (AMV RT), RNase H, and T7 DNA-dependent RNA polymerase (DdRp). The process starts with the reverse transcriptase enzyme synthesizing a cDNA strand using an RNA template, producing a hybrid RNA–DNA molecule. RNase H enzyme then degrades the RNA strand in the RNA–DNA hybrid molecule, leaving only the single-stranded DNA. T7 DdRp initiates transcription and synthesizes multiple RNA copies from the DNA template. These newly synthesized RNAs can serve as templates for further amplification. The amplification products can be detected using various methods, such as ethidium bromide (EtBr)-stained agarose gel electrophoresis, enzyme-linked gel assays, enzymatic bead-based detection, electrochemiluminescent detection, and fluorescent correlation spectroscopy. This technique offers several advantages over other mRNA amplification methods. Amplification of the RNA sequence (more than 10^9^ copies) can be performed within 90 min using the action of three enzymes. The amplification products can be directly used in another loop without denaturation or strand separation. The constant temperature allows each reaction step to proceed when an amplification intermediate becomes available [95]. On the other hand, the NASBA method has some limitations. Since the specificity of the reaction depends on heat-labile enzymes, the reaction temperature cannot exceed 42 °C without being affected. The length of the amplified RNA sequence should be between 120 and 250 nucleotides, as the amplification of shorter or longer sequences shows less efficiency [95]. 

An easy-to-operate G4-ThT fluorescent biosensor-NASBA system was developed to detect the classical swine fever virus (CSFV) [96]. This method could detect CSFV in serum samples within 2 h in real-time. It was reported that ThT reacted with amplification products to increase fluorescent responses, resulting in this system’s low LOD (2 copies/μL) in detecting CSFV. The optimized G4-ThT biosensor combined with NASBA offers numerous advantages, such as a rapid procedure, high sensitivity, economy, greater convenience, and broader application scenarios for detecting RNA viruses. This technique can be easily integrated into a highly automated system (e.g., microfluidic chip) owing to its real-time detection capability. 

Another study proposed a NASBA coupled with a CRISPR-Cas13a system to estimate the Salmonella virulence responsible for enteritis in mice [95]. This system allowed for sensitive (one colony-forming unit (CFU)) and rapid (within 2.5 h) detection of *S. enterica*. This assay could quantify viable *S. enterica* and improve the accuracy of virulence estimation compared to qPCR. A new variation of solid-phase NASBA was also introduced to amplify and detect RNA viruses. This method used surface-patterned biotinylated PEG microgels to tether amplification primers and molecular beacon detection probes in a spotted format appropriate for a microarray [95].

### 5.2. Helicase-Dependent Amplification

HDA is a molecular technique used for the detection of DNA and RNA viruses. This method is similar to PCR, but instead of heat denaturation, it utilizes helicase enzymes to separate RNA strands, which allows for RNA amplification without thermal cycling. The HDA reaction requires coordination between multiple replication proteins, including DNA helicase, DNA polymerase, and an optional ssDNA binding protein [97]. Although HDA was initially developed for DNA amplification, adaptations have been made for RNA virus detection. First, the RNA virus is typically reverse transcribed into complementary DNA (cDNA) before initiating the HDA process. The cDNA is then denatured to separate its strands. Primers specific to the target DNA sequence anneal to the single-stranded cDNA. Then, helicase enzymes, proteins obtained from thermophilic organisms, unwind the cDNA at high temperatures (60 to 65 °C) to allow the DNA polymerase to extend the primers, synthesizing new DNA strands [97]. The amplification process undergoes multiple cycles of denaturation, primer annealing, helicase activity, and extension, resulting in an exponential increase in DNA copies relative to the RNA in the sample. Finally, various methods can be used detect the amplification products, including fluorescent-based assays and gel electrophoresis [98,99,100]. The HDA technique offers potential advantages, such as rapid and sensitive detection of RNA viruses at a constant temperature without complex instrumentation. 

A POC technique combining RT-HDA with dipstick technology was developed to detect SARS-CoV-2 [98]. This diagnostic test was performed within a short period of about 2 h without any specialized equipment. The LOD of this diagnostics test was 6 copies/μL [98]. Another study compared the sensitivity of RT-HDA and RT-LAMP combined with a lateral flow assay to detect SARS-CoV-2 rapidly [19]. First, reverse transcription was performed using gene-specific primers at 42 °C for 30 min. The HDA reaction was then carried out at 65 °C for 90 min with a final volume of 50 μL. In addition, the RT-LAMP reaction was conducted with a final reaction volume of 25 μL at 65 °C for 60 min. The amplification results of the RT-HDA and RT-LAMP procedures were detected using lateral flow HybriDetect strips (Milenia Biotec, GieBen, Germany). The results showed that RT-HDA had an LOD of 3 copies/reaction for amplification conducted for 10–20 min, while the LOD of RT-LAMP was 30–300 copies/reaction within 40 min. The results suggested that RT-HDA combined with lateral flow was more sensitive than RT-LAMP. In addition, RT-HDA is inexpensive, simple, and practical, offering a potential alternative for developing POC tests for SARS-CoV-2 detection.

A colorimetric HDA method based on specific primers with HRPzyme was used to detect noroviruses in food samples [99]. RdRp and BPO genes were selected for the development of a colorimetric HDA. The LOD of this method was 10 copies/mL of each NoV GI and GII [99]. RT-HDA was also proposed to detect the tomato-spotted wilt virus (TSWV) in fifteen tissue types from suspected TSWV-positive plants [100]. The HDA amplification procedure was conducted at 65 °C within 60 min, showing a high specificity (4 pg RNA) compared with 40 pg RNA in the RT-PCR procedure. In addition, the HDA method showed a high sensitivity, specificity, and stability in detecting the Chinese sacbrood virus (CSBV) with an LOD of 10^−2^ ng [101]. The optimal reaction conditions were 65 °C, 90 min, and 5 μmol/L of primer. There was no cross-reaction with the cDNAs of healthy larvae, acute bee paralysis virus (ABPV), chronic bee paralysis virus (CBPV), black queen cell virus (BQCV), and deformed wing virus (DWV).

## 6. Commercialized Isothermal Amplification Devices for SARS-CoV-2 Detection

Several devices based on isothermal amplification techniques have been developed and commercialized for detecting SARS-CoV-2 (Table 4). These devices/kits can detect SARS-CoV-2 sensitively and rapidly without the use of complex equipment and handling procedures. These SARS-CoV-2 devices/kits are suitable for use in a variety of settings (e.g., hospitals and clinical laboratories) and play an important role in controlling the COVID-19 epidemic.

## 7. Limitations and Future Perspectives on Isothermal Amplification Methods

### 7.1. Recent Challenges of Isothermal Amplification Techniques

This review introduced some potential platforms that have integrated isothermal amplification for detecting RNA viruses. The device designs, types of isothermal amplification methods, and integration process of the methods into microfluidic platforms were described. However, the development of these devices presents several challenges. LAMP is one of the most common isothermal amplification methods because of its specificity and sensitivity. However, multiplex detection using LAMP can be challenging due to the complexity of the primer sets [102,103]. The design, ratios, and concentration of primer must also be carefully considered [102,103]. After amplification, post-processing is required to measure LAMP products, which increases the risk of cross-contamination or detection of non-specific LAMP amplicons [104]. Importantly, LAMP relies on indirect detection methods (e.g., turbidity and non-specific dyes), which might lead to false positive results [104]. LAMP products are large fragments, which limits downstream applications such as cloning [105].

RPA has its own set of challenges. False negative or false positive results may occur due to contaminants present during the assay. The high cost of RPA reagents can be a barrier, particularly when it comes to large-scale or routine testing. Although RPA assays are more affordable compared to alternative methods, their widespread adoption may be hindered by this issue [106]. Additionally, when RPA uses agarose gel electrophoresis to visualize amplification products, aerosolized DNA may escape, leading to cross-contamination after or during the opening of the reaction tubes [107]. These challenges must be considered when using the RPA method for RNA virus detection. 

The RAA method also has significant challenges that need to be overcome. RAA is highly sensitive, which can lead to false positive results. Therefore, it is crucial to prevent cross-contamination throughout the process. The short length of the target gene can produce nonspecific products during the reaction process [108]. Additionally, excessive primers and specific interactions between primer molecules can cause the formation of primer dimers, making it difficult to achieve on-site sample pre-treatment [109]. Another urgent challenge is achieving multi-target isothermal amplification detection under single-tube closed-tube conditions [84]. Without solutions to these issues, the range of applications for RAA may be limited. 

Similar to LAMP and RPA, HDA still faces important limitations, including sample contamination, non-specific amplification products causing false positives, and test inhibition leading to false negatives in complex biological matrices [110]. 

NASBA has a disadvantage, in that it requires the use of isothermal reaction thermolabile enzymes. Thus, the reaction must be carried out at a lower temperature than the PCR process, which uses thermocycling. As a result, there might be an increase in nonspecific primer interactions [111]. Moreover, NASBA is susceptible to contamination by extraneous nucleic acids, particularly its own amplification product carryover [111].

### 7.2. Future Perspectives

In order to improve the widespread applications of isothermal amplification, multiplex isothermal amplification systems have been proposed. LAMP is one of the most exploited isothermal techniques for multiplexing, despite the need for multiple primers. In a multiplex LAMP (mLAMP), a primer set that has four to six primers for each target is deposited in separate zones to prevent cross-reactivity [102]. Additionally, designing primers, maintaining ratios and concentrations, and using restriction enzymes are essential. For instance, an mLAMP method simultaneously detected bovine *Babesia* parasites using four specific primers, with a restriction enzyme cleavage site inserted into two pairs of species-specific primers. The amplicons were then differentiated via subsequent restriction enzyme analysis [112]. Similarly, a multiplex microfluidic LAMP could detect three influenza A subtypes in parallel with an LOD of less than 10 copies/μL within 30 min [113]. Another mLAMP assay successfully identified influenza A/H1, A/H3, and influenza B with an LOD of 1 genome equivalent. This assay utilized six primers to target the matrix genes with real-time fluorescent detection [114]. On the other hand, RPA requires a simple primer set, which is advantageous in multiplex and solid-phase amplification [102]. A solid-phase RPA amplification method (called on-chip asymmetric RPA) was developed for simultaneous detection of *Neisseria gonorrhoeae*, *Salmonella enterica*, and methicillin-resistant *Staphylococcus aureus* (MRSA) with an LOD of 10 copies of genomic DNA [115]. 

A rapid, sensitive, and specific POC method for the triplex detection of plant pathogens was generated using surface-enhanced Raman scattering (SERS) and RPA [116]. The assay exploited biotinylated reverse primes, tailed forward primers, and AuNPs functionalized with SERS nanotags and probed complementary to the tails of the primers. Furthermore, a few multiplexing systems have been reported using HDA. A thermophilic HDA (tHDA) could detect four targets, including two genes in *Chlamydia trachomatis*, a multicopy gene in *Neisseria gonorrhea*, and internal control. The reaction occurred at 65℃ for 90–120 min, with fluorescent detection [117]. Consequently, the use of multiplexed amplification and detection approaches will likely increase with the growing need for detecting multiple markers simultaneously.

Isothermal amplification is often plagued by the issue of generating unspecific products and background noise due to low amplification temperatures and non-specific amplification. Recent developments of background-free molecular circuits have been made to work in conjunction with isothermal amplification techniques, which can effectively reduce or eliminate non-specific signals or background noise [103,118]. This could be crucial for enhancing the sensitivity and specificity of the detection method. One proposed solution is a background-free isothermal amplification that relies on a DNA-based molecular method designed to catalyze a target-triggered signal amplification, preventing non-specific amplification reactions. This system used a leak absorption mechanism to suppress non-specific amplification, which is commonly encountered in other exponential amplification reactions [118]. With an estimated selectivity of over 97%, this approach showed promising results for microRNA digital assays [118]. 

Another group developed a test called CATCH, which used a synthetic gene circuit to detect ctDNA with high sensitivity and without background noise. CATCH combined a small transcription-activating RNA and a toehold switch in a multilevel switch to regulate transcription and translation simultaneously. This approach eliminated any background noise that may interfere with the accuracy of the test results [103]. To further suppress background amplification, a template lacking one of the four nucleotides (dT-free template), a three-letter nicking site, and 2′-deoxyriboadenosine 5′-triphosphate (dATP)-free amplification mixture was developed [119]. Peptide nucleic acid and locked nucleic acid modification of the 3′ domain of the EXPAR template have also been used to reduce background amplification [120,121]. 

Isothermal amplification approaches should be paired with other devices, such as CRISPR, to be more widely used in field applications. The integration of devices has been described in detail, but some platforms only perform nucleic acid amplification and detection. Additionally, sample preparation steps are still required to be conducted on-tube. The most significant challenge is the performance of the developed devices on real clinical samples. These issues limit the usefulness of the devices for real on-site detection. Therefore, more research is needed to optimize the full integration of sample preparation, nucleic acid amplification, and detection into one platform.

## 8. Conclusions

The widespread propagation of RNA viruses, particularly SARS-CoV-2, has urged the rapid evolution of diagnostic methods. These methods ensure a high accuracy, specificity, and sensitivity, a low cost, and simplicity for applications in low-resource areas. Nucleic acid amplification-based assays that detect the specific target genes are the gold standards for identifying RNA viruses. However, these methods usually require highly equipped laboratories, which limits their application in low-resource areas. Isothermal amplification is a promising alternative to conventional nucleic acid amplification methods. Moreover, isothermal amplification methods have been integrated into microfluidic platforms to realize rapid and POCT assays for RNA virus screening. This review introduced potential platforms that integrate isothermal amplification for detecting RNA viruses. The device designs, types of isothermal amplification methods, and integration process of the methods into microfluidic platforms were described. However, the development of these devices presents several challenges that limit their applications in real-time clinical sample analysis. Future studies should be conducted to address these issues and bring these devices from the bench to the bedside.

## Figures and Tables

**Table 1:** The current approaches using a LAMP-based microfluidic platform for RNA virus detection.

Type of Virus	Target	Sample	Detection Methods	Limit of Detection (LOD)	Time (min)	Temperature (°C)	Ref.
Ebola virus	ssRNA (−)	Cell culture	Nucleic acid strip detection (NAD)	30 copies/mL	35	58	[24]
African swine fever virus	dsRNA	Tissue	CRISPR-Cas12a nuclease reaction	7 copies/μL	30	37	[25]
HIV	ssRNA-RT	Plasma sample	CY5 channel fluorescence	89 copies/reaction	50	64	[26]
Tomato brown rugose fruit virus	ssRNA (+)	Tomato plants	Colorimetric assay	6 copies/μL	30	65	[27]
Mayaro virus	ssRNA (+)	Cell culture	Fluorescence-based point-of-care device	10 copies/reaction	30	60	[28]
Japanese encephalitis virus (JEV)	ssRNA (+)	Cell culture	Colorimetric assay	1 copy/μL	30	60	[29]
Zika virus	ssRNA (+)	Tissue culture	Fluorescence Genie III	6 copies/reaction	30	65	[30]
Zika virus	ssRNA (+)	Cell culture	Colorimetric-based toehold switch sensor	1.7 × 10^6^ copies/mL	30	37	[31]
HPV		Cervical swabs	Colorimetric-based microfluidic chip	50 copies/reaction	60	68	[32]
SAR-CoV-2 virus	ssRNA (+)	Nasopharyngeal aspirates and sputum samples	Colorimetric DNAzyme reaction	1 × 10^5^ copies/mL	30	65	[33]
SAR-CoV-2 virus	ssRNA (+)	Respiratory swab specimens	CRISPR-Cas12a-mediated fluorescence detection	30 copies/μL	40	60	[34]
SAR-CoV-2 virus	ssRNA (+)	Nasopharyngeal swabs	High-fidelity DNA polymerase-mediated fluorescence detection	115 copies/reaction	30	60	[35]
SAR-CoV-2 virus	ssRNA (+)	Nasopharyngeal and throat swabs	Cy5 channel fluorescence detection	20 copies/reaction	30	65	[36]
SAR-CoV-2 virus	ssRNA (+)	Nasopharyngeal, throat, and nose swabs	Lab-on-chip platform-based fluorescence detection	10 copies/reaction	30	63	[37]
SAR-CoV-2 virus	ssRNA (+)	Pharyngeal swab specimen	Colorimetric assay	30 copies/reaction	60	65	[38]
SAR-CoV-2 virus	ssRNA (+)	Clinical throat swab	Colorimetric and fluorescence assay	80 copies/mL	30	65	[39]
SAR-CoV-2 virus	ssRNA (+)	Nasopharyngeal swabs	Colorimetric and fluorescence assay	50 copies/reaction	31	65	[40]
SAR-CoV-2 virus	ssRNA (+)	Nasopharyngeal and throat swabs	Colorimetric-based electromechanical device	10^3^ copies/mL	30	65	[41]
SAR-CoV-2 virus	ssRNA (+)	Nasopharyngeal swabs	Colorimetric assay	0.75 copies/μL	55	65	[42]
SAR-CoV-2 virus	ssRNA (+)	Saliva samples	Fluorescence detection	5 copies/μL	45	65	[43]
SAR-CoV-2 virus	ssRNA (+)	Wastewater samples	Colorimetric-based electrochemical sensor	38 × 10^−6^ ng/μL	30	60	[44]

**Table 2:** Current approaches using an RPA-based microfluidic platform for RNA virus detection.

Type of Virus	Target	Sample	Detection Methods	LOD	Time (min)	Temperature (°C)	Ref.
Rabies virus	ssRNA (−)	Cell culture	IX51 Olympus fluorescence detection	100 copies/μL	30	50	[67]
Influenza A (H1N1) virus	ssRNA (−)	Cell culture	CRISPR-Cas12a nuclease reaction	10 copies/reaction	20	37	[68]
Influenza A (H7N9) virus	ssRNA (−)	Cell culture	Fluorescence detection	14 copies/μL	10	42	[61]
Influenza A and B	ssRNA (−)	Nasal fluid samples	Fluorescence-based H-sandwich detection	200 copies/mL	20	37	[66]
African swine fever virus (ASFV)		Swine sample	Glycerol-enhanced one-pot CRISP-Cas12a nuclease reaction	10 copies/μL	60	37	[69]
SAR-CoV-2 virus	ssRNA (+)	Respiratory swabs	CRISPR-Cas12a nuclease reaction	50 copies/μL	20	40	[70]
SAR-CoV-2 virus	ssRNA (+)	Respiratory swabs	Real-time fluorescence and dipstick detection	130 copies/reaction	30	39	[71]
SAR-CoV-2 virus	ssRNA (+)	Respiratory swabs	Cas12a nuclease reaction	6 copies/μL	30	37	[72]
SAR-CoV-2 virus	ssRNA (+)	Clinical throat swab	Lateral flow	100 copies/reaction	5	42	[73]
SAR-CoV-2 virus	ssRNA (+)	Clinical throat swab	CRISPR-Cas12a cleavage reaction and LED readout	20 copies/μL	20	37	[64]
Potato virus Y and S	ssRNA (+)	Cell culture	Nucleic acid lateral flow	5 × 10^9^ copies/reaction	30	37	[74]
HIV-1 virus	ssRNA (+)	HIV-1 integrated cells	Fluorescence-based microfluidic device	100 copies/mL	30	37	[75]
HIV	ssRNA (+)	HIV clinical samples	CRISPR-mediated cascade reaction biosensor using a glucose meter	43 copies/reaction	60	40	[76]
Citrus tristeza virus	ssRNA (+)	Plant samples	Lateral flow immunochromatographic assay	3.77 × 10^5^ copies/mL	20	40	[74]
Senecavirus A	ssRNA (+)	Piglet blood samples	Lateral flow dipstrip	15 copies/μL	25	35	[77]
Canine distemper virus	ssRNA (−)	Nasal/oropharyngeal swab	Real-time fluorescence detection	9.4 copies/μL	12	40	[78]
Chilli Veinal mottle virus	ssRNA (+)	Tobacco plant	Agarose gel electrophoresis	10 fg/μL	20	38	[79]
Wheat mosaic virus	ssRNA (+)	Wheat leaf	Agarose gel electrophoresis	10^−3^ ng/μL	20	45	[80]
Dengue virus	ssRNA (+)	Clinical specimens	Lateral flow dipstick	10 copies/μL	15	37	[81]

**Table 3:** Current approaches using RAA-based microfluidic platforms for RNA virus detection.

Type of Virus	Target	Sample	Detection Methods	LOD	Time (min)	Temperature (°C)	Ref.
Respiratory syncytial virus	ssRNA (−)	Nasopharyngeal aspirate	Real-time fluorescence detection	35 copies/reaction	30	39	[16]
Hepatitis D virus	ssRNA (+)	Plasma sample	Fluorescence and lateral flow strip	10 copies/μL	30	39	[89]
Coxsackievirus A10 and A6	ssRNA (+)	Clinical sample	Real-time fluorescence detection	35 copies/reaction	30	39	[85]
Japanese encephalitis virus	ssRNA (+)	Aborted fetuses and testicular swollen boars	Real-time fluorescence detection	5.5 copies/μL	30	39	[86]
Human norovirus	ssRNA (+)	Stool clinical samples	Fluorescence-based microfluidic chip	1.02 × 10^0^ copies/μL	20	39	[90]
SAR-CoV-2 virus	ssRNA (+)	Nasopharyngeal swabs	CRISPR-Cas13a electrochemical assay	1.66 × 10^1^ aM	30	42	[91]

**Table 4:** Commercialized isothermal products for COVID-19 screening.

Platform	Manufacturer	Assay	Specimen	Analysis Time	LOD
CBDNA RT-LAMP RAPID TEST	Centrum Badan DNA, Poland	RT-LAMP	Nasopharyngeal swab	20	10 copies/reaction
CBDNA RT-LAMP RAPID TEST	DNA Research Center Ltd., Poland	RT-LAMP	Nasal swab, nasopharyngeal swab, saliva, and throat swab	15	10 copies/reaction
GenomtecSARS-CoV-2 EvaGreen^®^ RT-LAMP CE-IVD Duo Kit	Genomtec SA, Poland	RT-LAMP	Nasopharyngeal swab, oropharyngeal swab, and saliva	30	20 copies/reaction
SARS-CoV-2 EvaGreen^®^ Direct-RT-LAMP CE-IVD Kit	Genomtec SA, Poland	RT-LAMP	Nasopharyngeal swab, oropharyngeal swab, and saliva	40	2 copies/reaction (saliva)10 copies/reaction (dry swab)
GENEDIA W COVID-19 Colorimetric LAMP premix kit	Green Cross Medical Science Corp., South Korea	RT-LAMP	Nasopharyngeal swab and oropharyngeal swab	32	100 copies/reaction
Hayat Rapid Colorimetric & Fluorimetric One Step LAMP SARS-CoV-2 Test Kit	Hayat Genetics Inc., Turkey	RT-LAMP	Nasal swab, nasopharyngeal swab, and saliva	30	5 copies/reaction
COVID-19 Nucleic Acid Detection Kit (RT-LAMP)	Langfang Xinruikang Biotechnology Co. Ltd., China	RT-LAMP	Anterior nasal swab, nasopharyngeal swab, and oropharyngeal swab	30	500 counts/min
Vivid COVID-19 LAMP Direct-G	MultiplexDX s.r.o., Slovakia	RT-LAMP	Biological fluids, sputum, throat secretion	40	1000 counts/min
LAMPIGEN COVID19 RT-LAMP PCR KIT	Pharmaline AS, Turkey	RT-LAMP	Anterior nasal swab, nasal swab, nasopharyngeal swab, and throat swab	30	3 copies/μL
1copy™ COVID-19 MDx Kit Professional	1drop Inc., South Korea	RT-LAMP	Nasopharyngeal swab	20	100 copies/reaction
Atila iAMP COVID-19 SANO Assay	Atila BioSystems Inc., United States	RT-LAMP	Nasopharyngeal swab, oropharyngeal swab, saliva	70	AU 2.5
BiologyWorks	BiologyWorks Inc., United States	LAMP	Anterior nasal swab, nasal swab	45	30,000 counts/min
C4Covid-19 Human	C4Diagnostics, France	RT-LAMP	Saliva	30	210,000 AU
DigiGENE™ COVID-19 Integrated Molecular Test	Canary Global Inc., Canada	RT-LAMP	Mid-turbinates swab, oropharyngeal swab	20	600 counts/min
SARS-CoV-2 Nucleic Acid Detection Kit	CapitalBio Technology, China	RT-LAMP	Nasal swab, nasopharyngeal swab, throat swab	45	150 counts/min
Dotz SARS-CoV-2 Rapid Diagnostic Kit	Dotz Nano Ltd., Israel	RT-LAMP	Nasopharyngeal swab, oropharyngeal swab, saliva	34	1250 copies/reaction
SARS-CoV-2 POC	Enbiotech s.r.l, Italy	RT-LAMP	Nasopharyngeal swab, oropharyngeal swab	60	50 copies/reaction
COV19-ID Kit	Genedrive, United Kingdom	RT-LAMP	Nasal swab	17	500 counts/min
LoopDeetect COVID 19 IC	LoopDeeScience, France	RT-LAMP	Anterior nasal swab, nasal swab, nasopharyngeal swab, oropharyngeal swab	45	-
MD-Bio BCC19 Test Kit	MD-Bio Inc., United States	RT-LAMP	Nasal swab, nasopharyngeal swab	30	75%
NaorCov19	Rapid Diagnostic Systems Limited, Israel	RT-LAMP	Saliva	40	40 copies/μL
Multitest COVID-19	Selfdiagnostics Deutschland GmbH, Germany	RT-LAMP	Anterior nasal swab	45	**-**
Dr Vida pocket for COVID-19	STAB VIDA, Portugal	RT-LAMP	Nasopharyngeal swab	40	75 particles
Vicare Rapid FL	Vicare Solution GmbH, Germany	RT-LAMP	Deep (cough) sputum, nasal swab, nasopharyngeal swab, oropharyngeal swab, saliva, sputum	30	150 cpu
SARS-CoV-2 Isothermal Amplification Detection Kit	Xiamen Jiqing Biomedical Technology Co. Ltd., China	RT-LAMP	Nasal swab, throat swab	30	500 counts/min
ID NOW^TM^ COVID-19	Abbott	NEAR	nasopharyngeal swab	15	125 counts/min
Lucira COVID-10 All-In-One Test Kit	Lucira Health	RT-LAMP	Nasal swab	30	900 counts/min
POA-nCiVn-LFD-16 ket	BioPOA Co., Ltd. Korea	RPA	Nasopharyngeal swab and oropharyngeal swab	20	20 copies/reaction

## Data Availability

The data is not available.
